# Diminazene Aceturate Stabilizes Atherosclerotic Plaque and Attenuates Hepatic Steatosis in apoE-Knockout Mice by Influencing Macrophages Polarization and Taurine Biosynthesis

**DOI:** 10.3390/ijms22115861

**Published:** 2021-05-30

**Authors:** Aneta Stachowicz, Anna Wiśniewska, Katarzyna Kuś, Magdalena Białas, Magdalena Łomnicka, Justyna Totoń-Żurańska, Anna Kiepura, Kamila Stachyra, Maciej Suski, Beata Bujak-Giżycka, Jacek Jawień, Rafał Olszanecki

**Affiliations:** 1 Chair of Pharmacology, Jagiellonian University Medical College, 31-531 Krakow, Poland; anna.niepsuj@uj.edu.pl (A.W.); katarzyna.1.kus@uj.edu.pl (K.K.); m.lomnicka@uj.edu.pl (M.Ł.); justyna.toton-zuranska@uj.edu.pl (J.T.-Ż.); a.kiepura@uj.edu.pl (A.K.); kamila.stachyra@uj.edu.pl (K.S.); maciej.suski@uj.edu.pl (M.S.); beata.bujak-gizycka@uj.edu.pl (B.B.-G.); jacek.jawien@uj.edu.pl (J.J.); rafal.olszanecki@uj.edu.pl (R.O.); 2 Chair of Pathomorphology, Jagiellonian University Medical College, 31-531 Krakow, Poland; magdalena.bialas@uj.edu.pl

**Keywords:** atherosclerosis, fatty liver, DIZE, macrophages polarization, apoE-knockout mice, ACE21

## Abstract

Atherosclerosis and nonalcoholic fatty liver disease are leading causes of morbidity and mortality in the Western countries. The renin–angiotensin system (RAS) with its two main opposing effectors, i.e., angiotensin II (Ang II) and Ang-(1–7), is widely recognized as a major regulator of cardiovascular function and body metabolic processes. Angiotensin-converting enzyme 2 (ACE2) by breaking-down Ang II forms Ang-(1–7) and thus favors Ang-(1–7) actions. Therefore, the aim of our study was to comprehensively evaluate the influence of prolonged treatment with ACE2 activator, diminazene aceturate (DIZE) on the development of atherosclerotic lesions and hepatic steatosis in apoE^−/−^ mice fed a high-fat diet (HFD). We have shown that DIZE stabilized atherosclerotic lesions and attenuated hepatic steatosis in apoE^−/−^ mice fed an HFD. Such effects were associated with decreased total macrophages content and increased α-smooth muscle actin levels in atherosclerotic plaques. Moreover, DIZE changed polarization of macrophages towards increased amount of anti-inflammatory M2 macrophages in the atherosclerotic lesions. Interestingly, the anti-steatotic action of DIZE in the liver was related to the elevated levels of HDL in the plasma, decreased levels of triglycerides, and increased biosynthesis and concentration of taurine in the liver of apoE^−/−^ mice. However, exact molecular mechanisms of both anti-atherosclerotic and anti-steatotic actions of DIZE require further investigations.

## 1. Introduction

Atherosclerosis and nonalcoholic fatty liver disease (NAFLD) are leading causes of morbidity and mortality in the Western countries [1]. Atherosclerosis, a multifactorial inflammatory illness of arteries, is characterized by a lipid-rich necrotic core and a rupture-prone fibrous cap. The rupture of the atherosclerotic plaque can cause life-threatening complications: coronary artery disease, stroke, peripheral artery disease, and myocardial infarction [2]. Endothelial activation is an initial step of atherogenesis that promotes the accumulation of oxidized low-density lipoproteins (ox-LDL), monocytes, and other inflammatory cells in the subendothelial space. Subsequently, the engulfment of ox-LDL by macrophages, exacerbation of inflammation, migration and activation of vascular smooth muscle cells (VSMC), and finally, apoptosis of macrophages and VSMC occurs [3,4]. Mounting evidence indicates that NAFLD, which is manifested by triglyceride accumulation in the hepatocytes, is an important independent risk factor for atherogenesis [5]. It encompasses a variety of pathological conditions, such as simple hepatic steatosis, steatosis with inflammatory response—nonalcoholic steatohepatitis (NASH), cirrhosis and fibrosis, and hepatocarcinoma [6]. The pathogenesis of NAFLD could be described by classical “two hit-hypothesis”, where initial lesions in the liver are caused by excess accumulation of free fatty acids, while further damage and subsequent inflammation and fibrosis are triggered by oxidative stress and proinflammatory cytokines [7]. The renin–angiotensin system (RAS) is widely recognized as a major regulator of cardiovascular function and body metabolic processes [8,9]. The classical axis of RAS, i.e., angiotensin-converting enzyme (ACE)/Ang II/AT1 has been shown to contribute to the development of atherosclerosis and NAFLD [10,11]. Angiotensin II (Ang II), which is a product of the conversion of Ang I by ACE, has pro-oxidant, proinflammatory, and prothrombotic properties. It increases vascular permeability, oxidation and uptake of LDL, inflammatory cell infiltration, and generation of reactive oxygen species [12,13]. Ang II action is counteracted by Ang-(1–7), which is produced from Ang II by ACE2. It has been reported that ACE2/Ang-(1–7)/Mas axis had atheroprotective effects as well as inhibited hepatic insulin resistance, improved glucose uptake, and decreased glycogen synthesis [14,15]. Indeed, overexpression of ACE2 attenuated atherosclerosis and enhanced atherosclerotic plaque stability in a rabbit model of atherogenesis, and contrarily, its genetic deficiency worsened atherosclerosis in apoE-knockout mice [16,17]. Additionally, deletion of ACE2 in mice aggravates hepatic steatosis, inflammation, and oxidative stress [18]. Taking into account the role of ACE2 in cardiovascular and metabolic processes, its pharmacological activation might have the beneficial effects in the treatment of atherosclerosis and NAFLD. In 2011, it was shown that diminazene aceturate (DIZE) has an ability to increase ACE2 activity. DIZE is an aromatic diamidine approved by the US Food and Drug Administration for the treatment of human trypanosomiasis, but almost for six decades, it is used mainly as an antitrypanosomal drug in animals. The drug is well known and devoid of major toxic effects, thus may be a good candidate for repurposing [19]. Noteworthy, it has been shown that DIZE could attenuate pulmonary hypertension, myocardial infarction, and type 1 diabetes and reduce adiposity [20,21,22]. DIZE was recently shown to attenuate post-myocardial infarction contractile and electrophysiological dysfunction [23]. Interestingly, DIZE has been also proposed as a potential drug to prevent novel severe acute respiratory syndrome coronavirus 2 (SARS-CoV-2) complications. All these data make DIZE an interesting drug candidate with new indications.

Apolipoprotein E-knockout (apoE^−/−^) mice that spontaneously develop atherosclerotic lesions, hypercholesterolemia, and dyslipidemia are a popular animal model of atherosclerosis. Along with atherosclerotic plaques, they also exhibit mild hepatic steatosis, which is more exacerbated in mice on a high-fat diet. Thus, the aim of our study was to comprehensively evaluate the influence of prolonged treatment with ACE2 activator, diminazene aceturate (DIZE), on the development of atherosclerotic lesions and hepatic steatosis in apoE^−/−^ mice fed a high-fat diet (HFD).

## 2. Results

### 2.1. Influence of DIZE on Atherosclerosis Progression

**Figure 1:**
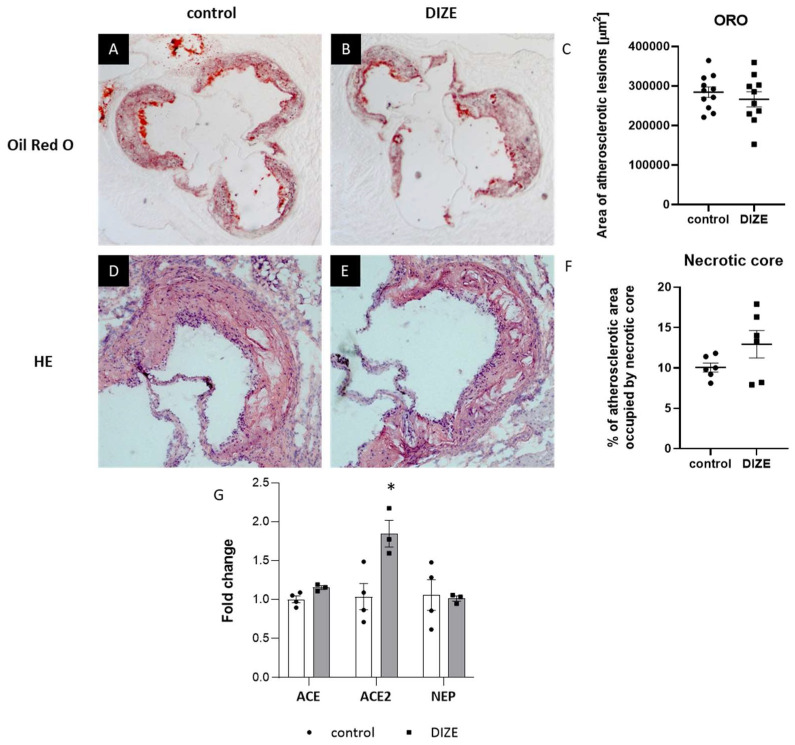
Influence of DIZE on atherosclerosis progression. Representative micrographs showing oil-red O-stained atherosclerotic lesions (**A**,**B**) and HE-stained necrotic cores (**D**,**E**) in the aorta of control and DIZE-treated mice as well as their corresponding quantitative analyses (**C**,**F**). mRNA expression of ACE, ACE2, and NEP in the aorta of control and DIZE-treated mice (**G**). Data are mean ± SEM analyzed using *t*-test (* *p* < 0.05 as compared to control; *n* = 3–11 per group).

**Figure 2:**
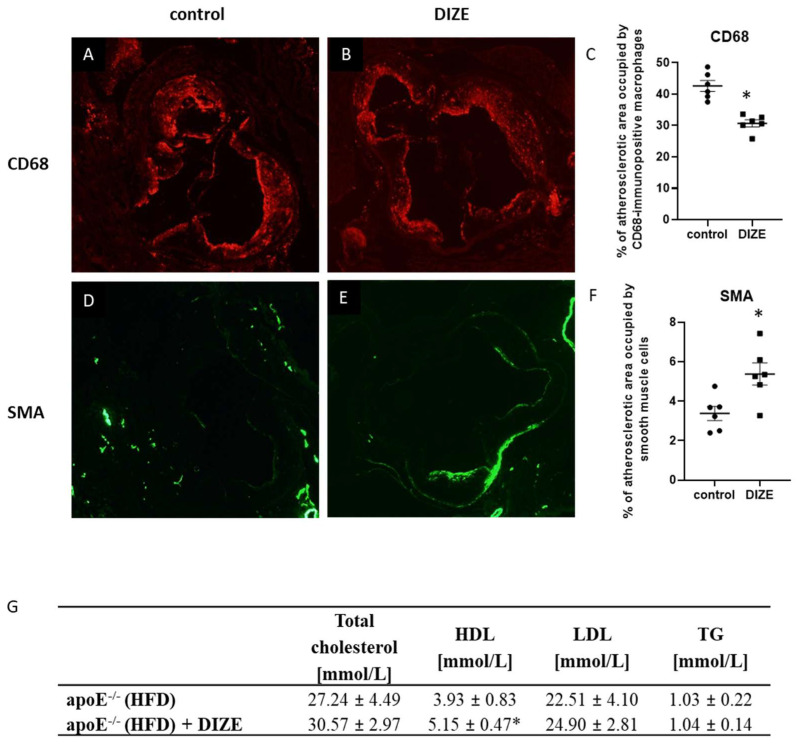
Impact of DIZE on the stability of atherosclerotic plaques. Immunohistochemical staining of aortic roots showing CD68-positive macrophages (**A**,**B**) and smooth muscle α-actin (SMA) (**D**,**E**) in control and DIZE-treated mice. Quantitative analysis of the atherosclerotic lesions area occupied by CD68-positive macrophages (**C**) and smooth muscle cells (**F**). The plasma levels of total cholesterol, high-density lipoproteins (HDL), low-density lipoproteins (LDL), and triglycerides (TG) (**G**) in control and DIZE-treated mice. Data are mean ± SEM analyzed using *t*-test (* *p* < 0.05 as compared to control mice; *n* = 4 or 6 per group).

To evaluate the impact of DIZE on the development of atherosclerosis, we treated apoE^−/−^ mice fed a high-fat diet with DIZE (30 mg per kg of body weight per day) for 16 weeks. The treatment neither caused significant decrease in atherosclerotic lesions in the aorta of apoE^−/−^ mice as measured by “cross-section” method (266,550 ± 19,271 vs. 284,551 ± 13,070 μm^2^; *p* > 0.05) (Figure 1A–C) nor reduced the necrotic core in atherosclerotic plaques (12.9% ± 1.5% vs. 10.1% ± 0.6%; *p* < 0.05) (Figure 1D–F). However, DIZE administration stabilized atherosclerotic lesions in apoE^−/−^ mice: it significantly decreased the macrophages content as evidenced by CD68 staining (30.7% ± 1.1% vs. 42.6% ± 1.7%; *p* < 0.05) (Figure 2A–C) and increased the smooth muscle α-actin (SMA) content (5.4% ± 0.6% vs. 3.4% ± 0.4%; *p* < 0.05) (Figure 2D–F). It seems that DIZE action was associated with increased mRNA expression of ACE2 enzyme, but not ACE and neprilysin (NEP) enzymes, in the aorta of apoE^−/−^ mice (Figure 1G).

**Figure 3:**
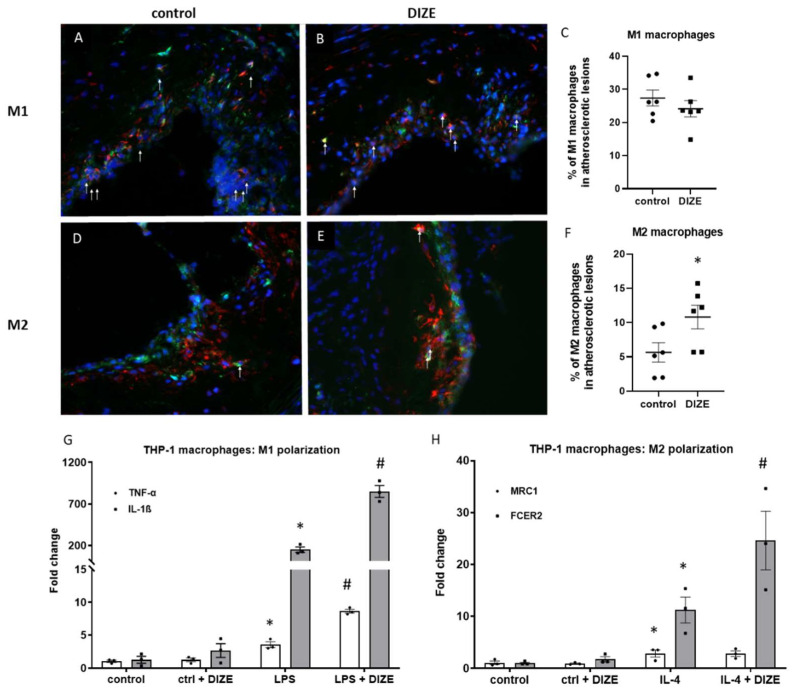
Macrophages polarization in atherosclerotic lesions and THP-1 cell culture after treatment with DIZE. Representative immunohistochemical staining of aortic roots showing F4/80 (green), nitric oxide synthase 2 (iNOS)/arginase 1 (red), and 4′6-diamidino-2-phenylindole (DAPI) (blue) co-localization in control (**A**,**D**) and DIZE-treated mice (**B**,**E**). White arrows indicate M1 (**A**,**B**) and M2 (**D**,**E**) macrophages, respectively. Quantitative analysis of M1 and M2 contents in the atherosclerotic plaques (**C**,**F**). mRNA expression of M1 (IL-1β and TNF-α) (**G**) and M2 (MRC1, FCER2) (**H**) markers in THP-1 macrophages cell culture polarized to proinflammatory M1 and anti-inflammatory M2 phenotype after treatment with DIZE. Data are mean ± SEM analyzed using *t*-test (**C**,**F**) or one-way ANOVA with multiple comparisons and Benjamini and Hochberg false discovery rate (FDR) correction (**G**,**H**) (* *p* < 0.05 as compared to control; ^#^ *p* < 0.05 as compared to LPS or IL-4, respectively; *n* = 3 independent experiments or *n* = 6 biological replicates per group).

To further explore the reduced number of macrophages after DIZE administration, we checked whether DIZE can change the content of proinflammatory M1 and anti-inflammatory M2 phenotypes of macrophages in atherosclerotic plaques. Interestingly, treatment with DIZE led to the elevated level of M2 macrophages (10.8% ± 1.7% vs. 5.7% ± 1.4%; *p* < 0.05) (Figure 3A–C) in atherosclerotic lesions of apoE^−/−^ mice, but did not change the content of M1 macrophages (24.1% ± 2.5% vs. 27.4% ± 2.4%; *p* > 0.05) (Figure 3D–F). To confirm those results, we performed in vitro experiments of polarization of THP-1 macrophages to M1 and M2 phenotypes in presence of DIZE (10 μM). Indeed, DIZE treatment led to 2 times increased level of the one of anti-inflammatory M2 markers (Fc Fragment of IgE Receptor II, FCER2) in THP-1 macrophages stimulated with IL-4 (Figure 3H). Surprisingly, it also significantly elevated mRNA expression of proinflammatory M1 markers (IL-1β and TNF-α) in THP-1 macrophages stimulated with LPS (Figure 3G).

### 2.2. Influence of DIZE on Mesenteric Arteries Responses Ex Vivo

**Figure 4:**
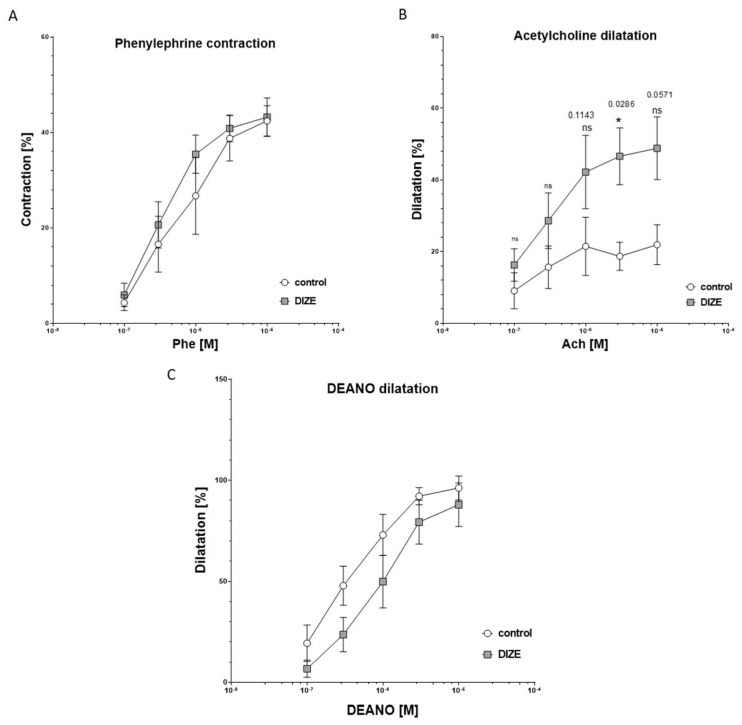
Graphs showing the time course response of mesenteric arteries to phenylephrine (**A**), acetylcholine (**B**), and DEA-NO (**C**) from control and DIZE-treated mice. Data are mean ± SEM analyzed using nonparametric Mann–Whitney U test (* *p* < 0.05 as compared to control mice; *n* = 4–6 per group).

We also checked the effect of DIZE on mesenteric arteries from intestine. There was no difference between DIZE-treated mice and controls regarding contraction of mesenteric arteries induced by phenylephrine (Figure 4A). Similarly, relaxations to endothelium-independent vasodilator DEA-NO did not differ between groups (Figure 4C). However, DIZE slightly increased maximal dilatation induced by acetylcholine at the highest concentrations of Ach (Figure 4B). Besides, Ang II induced two-phase response: contractions at lower concentration and relaxation at higher, but those differences were not significant between DIZE and the control group. KCl only at lowest doses of 30 mM induced less contraction in arteries of mice from DIZE group, but those differences declined at higher concentrations. Moreover, EC50 did not change significantly between groups.

### 2.3. Influence of DIZE on Hepatic Steatosis

**Figure 5:**
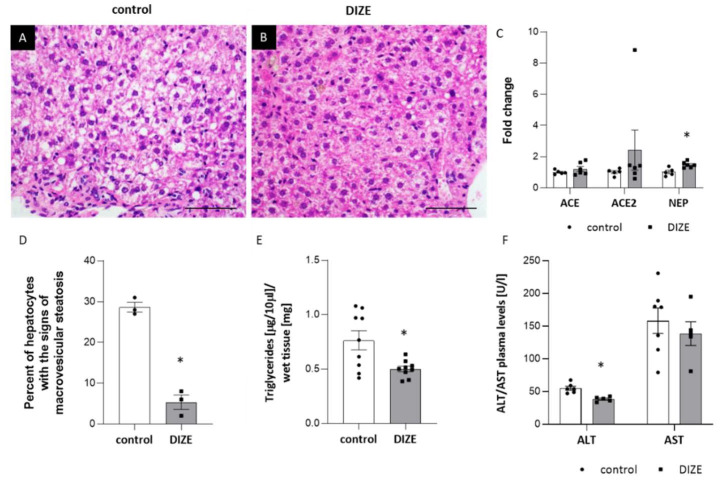
Influence of DIZE on the development of hepatic steatosis. Representative images of livers in con-trol (**A**) and DIZE-treated mice (**B**). The figures show hematoxilin and eosin staining (**A**,**B**) and quantitative analysis of macrovesicular steatosis (**D**), triglycerides content in the liver (**E**) as well as plasma ALT/AST levels (**F**) in control and DIZE-treated mice. mRNA expression of ACE, ACE2 and NEP in the liver of control and DIZE-treated mice (**C**) Magnification 40×. Data are mean ± SEM analyzed by using *t*-test (* *p* < 0.05 as compared to control mice; *n* = 3–9 per group).

To evaluate the impact of DIZE on the development of hepatic steatosis in the liver of apoE^−/−^ mice, we used hematoxylin/eosin (HE) staining. The cytoplasm of hepatocytes had a granular structure with signs of macrovesicular steatosis of about 28% of hepatocytes present in all three lobular zones, and treatment with DIZE reduced it to about 5% of hepatocytes, mostly in the first zone (Figure 5A,B,D). Furthermore, DIZE administration resulted in the significant decrease in level of triglycerides by about 33% in the liver, but not in the plasma of apoE^−/−^ mice (Figure 2G and Figure 5E). However, it increased the content of high-density lipoproteins (HDL) in the plasma of apoE^−/−^ mice (Figure 2G). In addition, DIZE treatment lowered plasma level of the one of the markers of liver damage: alanine aminotransferase (ALT) (Figure 5F). It seems that DIZE action was associated with increased mRNA expression of NEP enzyme and tended to augment mRNA expression of ACE2 in the liver of apoE^−/−^ mice (Figure 5C).

### 2.4. Influence of DIZE on Proteomic Changes in the Liver

**Figure 6:**
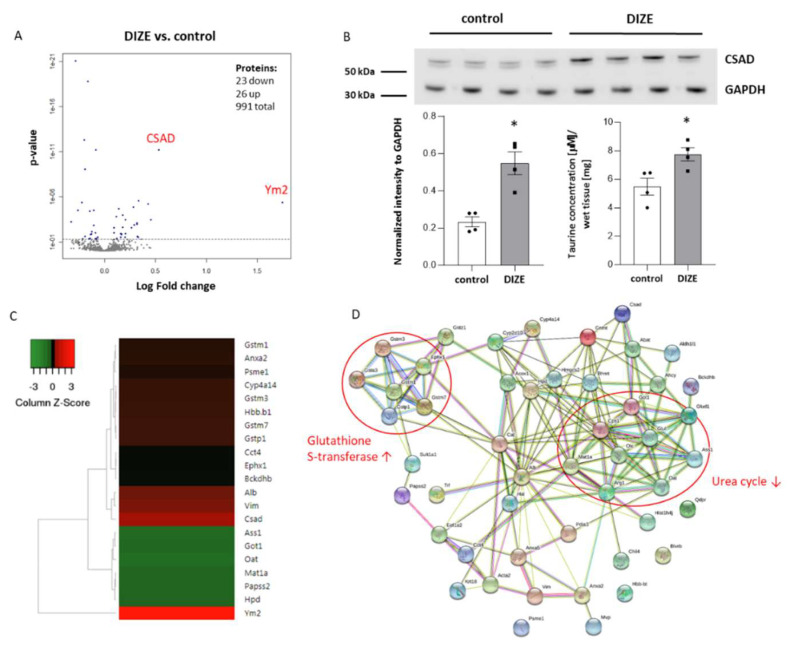
Proteomic analysis in the liver of DIZE-treated mice. The volcano plot of differentially expressed proteins showing the log2 fold change of protein expression vs. *p*-value in DIZE group compared to control mice (**A**). Validation of cysteine sulfinic acid decarboxylase (CSAD) expression by Western blot as well as the taurine levels in the liver of DIZE-treated mice (**B**). Heat map presentation of a hierarchical cluster of significantly changed proteins in the liver of DIZE-treated mice (**C**) (fold change >1.15 and <−1.15). Bioinformatic analysis by STRING of differentially expressed proteins reveals decreased expression of urea cycle proteins and increased expression of different isoforms of glutathione S-transferase (**D**). Data are mean ± SEM analyzed using *t*-test (**B**) or one-way ANOVA with Benjamini and Hochberg false discovery rate (FDR) correction (**A**,**C**) (* *p* < 0.05 as compared to control mice; *n* = 4 per group).

To further explore the beneficial effect of DIZE administration on the reduction in hepatic steatosis in apoE^−/−^ mice, we used proteomic methods. Isobaric tag for relative quantitation (iTRAQ method) combined with the multiple enzyme digestion filter aided by a sample preparation method (MED FASP) and LC-MS analysis discovered 49 differentially expressed proteins in the liver of apoE^−/−^ mice after treatment with DIZE (Table 1). The results were presented as either volcano plot based on log_2_ fold change and *p*-value (Figure 6A) or heat map, which shows the most differentially expressed proteins in the liver of DIZE-treated apoE^−/−^ mice (Figure 6C). The most upregulated proteins were chitinase-like protein 4 (Ym2, fold change 3.36) and cysteine sulfinic acid decarboxylase (CSAD, fold change 1.45). The increased expression of CSAD in the liver of DIZE-treated apoE^−/−^ was also confirmed by Western blot (Figure 6B). As CSAD is an enzyme participating in taurine biosynthesis, we checked the level of taurine in the liver of apoE^−/−^ mice as well. Indeed, DIZE-treated apoE^−/−^ mice had higher concentration of taurine in the liver in comparison to control mice (Figure 6B). In addition, DIZE administration led to the decreased expression of protein related to urea cycle: ornithine carbamoyltransferase, carbamoyl-phosphate synthase, arginase-1, aspartate aminotransferase, argininosuccinate synthase, ornithine aminotransferase as well as increased expression of different subunits of glutathione S-transferase (P 1, Mu 7, Mu 3, Mu 1) (Figure 6D) in the liver of apoE^−/−^ mice.

## 3. Discussion

The renin–angiotensin system (RAS) with its two main opposing effectors: Ang II and Ang-(1–7) is widely recognized as a major regulator of cardiovascular function and body metabolic processes [8,9]. ACE2 by breaking-down Ang II forms Ang-(1–7) and thus favors Ang-(1–7) actions. In the present study, we found that ACE2 activator, diminazene aceturate (DIZE), stabilized atherosclerotic plaque and attenuated hepatic steatosis in apoE^−/−^ mice by influencing macrophages polarization and taurine biosynthesis.

ACE2/Ang-(1–7)/Mas axis has been shown to elicit atheroprotective effects [14]. Genetic knockdown of ACE2 worsened atherosclerosis in apoE^−/−^ and low-density lipoprotein receptor (Ldlr ^−/−^) knockout mice and consistently, its overexpression attenuated atherosclerosis and enhanced atherosclerotic plaque stability [16,17,24]. Interestingly, our results indicate that even prolonged administration of DIZE mixed with an HFD diet at a dose of 30 mg/kg/day for 16 weeks did not reduce atherosclerotic lesions and necrotic core in apoE^−/−^ mice. It is in line with other study showing that shorter treatment with DIZE at a lower dose of 15 mg/kg/day for 3 weeks did not attenuate atherogenesis but stabilized atherosclerotic lesions in a shear stress-induced model of vulnerable atherosclerotic plaque in apoE^−/−^ mice [25]. Indeed, our results also point out stabilization of atherosclerotic lesions after prolonged DIZE treatment: decreased macrophages content as evidenced by CD68 staining and increased smooth muscle α-actin (SMA) content. This is consistent with the previous studies showing stabilized atherosclerotic lesions in animal models of either overexpressed ACE2 or pharmacologically activated ACE2, with reduced inflammatory cells infiltration as well as MMP-9 and MMP-3 levels, and increased collagen content [16,25].

To more deeply explore the composition of atherosclerotic plaques after DIZE administration, we also checked the content of proinflammatory M1 and anti-inflammatory M2 phenotypes of macrophages. Interestingly, DIZE treatment elevated the level of M2 macrophages but did not change the level of M1 macrophages in atherosclerotic lesions of apoE^−/−^ mice, which might further indicate more stabilized atherosclerotic plaques after prolonged DIZE administration. Tissue-resident macrophages elicit vast plasticity and can be classified according to two main phenotypes: proinflammatory M1 macrophages, characterized by production of nitric oxide (NO) and inflammatory cytokines (IL-1β and TNF-α) and being responsible for the clearance of pathogens, and anti-inflammatory M2 macrophages, which release an anti-inflammatory cytokine IL-10 and play a role in resolution of inflammation, tissue repair, and wound healing [26,27]. Importantly, it has been shown that macrophage phenotype may have impact on plaque vulnerability in atherosclerosis. More M1 macrophages were observed in unstable plaque and more M2 macrophages were seen in stable plaque in patients with acute ischemic attack [28]. In addition, several studies have demonstrated that M1 macrophage phenotype is linked to atherosclerosis progression [29,30] and, in turn, the induction of macrophage polarization to M2 by IL-13 could reduce disease progression [31]. Therefore, the macrophage class switching to M2 phenotype induced by DIZE could be a potential therapeutic approach in the treatment of atherosclerosis.

Moreover, we confirmed macrophage polarization results in vitro using a cell line model: THP-1 macrophages polarized to M1 and M2 in the presence of DIZE. In fact, DIZE administration increased level of anti-inflammatory M2 marker (FCER2) in THP-1 macrophages differentiated to M2 phenotype. Surprisingly, however, it also elevated gene expression of proinflammatory M1 markers (IL-1β and TNF-α) in THP-1 macrophages polarized to M1. Discrepancies between in vivo and in vitro results of M1 macrophage levels after DIZE treatment might be due to the presence and the role of membrane bound ACE2 vs. soluble circulating form of ACE2, which is a proteolytic product of shedding of membrane-bound ACE2 by disintegrin and metalloproteinase domain-containing protein 17 (ADAM17) [32]. Nevertheless, our results are in contrast with other study showing that DIZE suppressed the production of proinflammatory cytokines: IL-6, TNF-α, and IL-12 in bone marrow-derived macrophages (BMDM) and mice challenged with LPS [33]. Further research is needed to clarify the impact of ACE2 activator, DIZE, on proinflammatory macrophages, especially nowadays, as ACE2 is recognized as both binding receptor for severe acute respiratory syndrome coronavirus 2 (SARS-CoV-2) and important factor limiting lung injury in coronavirus disease-19 (COVID-19) caused by SARS-CoV-2. Only recently, DIZE has been proposed as a potential drug to prevent SARS-CoV-2 complications [34].

A growing body of evidence indicates that NAFLD is an important independent risk factor for the development of atherosclerosis [5]. ACE2/Ang-(1–7)/Mas axis has been reported to contribute to the development of NAFLD [15]. In this study, we showed that ACE2 activator, DIZE, attenuated hepatic steatosis in apoE^−/−^ mice along with the reduction in triglycerides content in the liver and upregulation of HDL level in the plasma. In addition, DIZE improved liver function by decreasing the level of alanine aminotransferase (ALT). Our results are in line with other studies showing that genetic knockdown of ACE2 in mice aggravated hepatic steatosis, oxidative stress, and inflammation by activating Akt signaling [18]. In turn, oral administration of Ang-(1–7) prevented hepatic steatosis, improved metabolism, and decreased inflammation in mice [35]. Moreover, transgenic rats overexpressing Ang-(1–7) had lowered level of triglycerides in the liver [36].

To elucidate the mechanism of action of DIZE in the liver of apoE^−/−^ mice, we applied proteomic method: iTRAQ combined with the multiple enzyme digestion filter aided by a sample preparation method (MED FASP) and LC-MS analysis. Among 49 differentially expressed proteins, two proteins with the highest upregulation level after DIZE treatment: chitinase-like protein 4 (Ym2) and cysteine sulfinic acid decarboxylase (CSAD) are worth further discussion. Ym2 belongs to the glycoside hydrolase family 18 of proteins that are responsible of chitin degradation and act as host-defense enzymes. Little is known about Ym2 function, as this protein is less abundant, was not widely studied and has high sequence similarity (∼95%) to Ym1 [37]. Ym1 is considered as an M2 marker in mouse and may play a role in inflammatory responses and allergy [38]. However, whether DIZE not only increases the content of M2 macrophages in the atherosclerotic lesions but also in the liver requires further investigations. In turn, CSAD is an enzyme participating in taurine biosynthesis, which converts cysteine sulfinic acid to hypotaurine and CO_2_. Our proteomics results showed increased expression of CSAD in the liver of DIZE-treated apoE^−/−^ mice, which was additionally confirmed by Western blot. Consistently, we also observed elevated taurine concentration in the liver of apoE^−/−^ mice after DIZE administration. Taurine is one of the most abundant amino acids in mammals and basic regulator of biological and physiological processes. It has been shown that taurine could prevent atherogenesis in mice and rabbits by influencing osmoregulation, oxidation, and inflammation [39] as well as could attenuate hepatic steatosis in mice on an HFD through the inhibition of oxidative stress [40]. Interestingly, taurine has been also reported to modulate the phenotype of macrophages towards increasing M2 macrophages in adipose tissues, which was measured by elevated gene expression of M2 markers: Ym1, Arg1, and MGL1 [41]. Thus, increased biosynthesis and concentration of taurine in the liver of apoE^−/−^ mice treated with DIZE might be presumably one of the beneficial mechanisms of DIZE action in the reduction in hepatic steatosis and stabilization of atherosclerotic plaques.

In our setting, the use of DIZE did not change the response of the mesenteric arteries to phenylephrine and the NO donor (DEA-NO) but increased the endothelial-dependent relaxation induced by acetylcholine. The mechanism of such effect could depend on an increase in endothelial eNOS-derived NO release and/or improvement of NO bioavailability. Interestingly, DIZE has recently been shown to increase NO production in the mesenteric artery of SHR-treated rats, but the mechanism of this action has not been thoroughly characterized [42]. Intriguingly, recently NO secretion-enhancing effect of taurine has been described [43]. Several possible mechanisms of such action of taurine have been proposed, i.e., increasing eNOS expression, eNOS phosphorylation on Ser1177, NO bioavailability, the level of antioxidative defense, and the influence on L-arginine/NOS inhibitor asymmetric dimethylarginine (ADMA) ratio, however, whether and which of them may be involved in the action of DIZE in our experimental model requires further research.

Our research has several strengths: we investigated a compound with a low, well-established toxicity that is well suited to repurposing and new use. We also pointed out new, interesting mechanisms of the drug’s action that may be responsible for the stabilization of atherosclerotic plaque and the reduction in fatty liver. It is tempting to speculate that ACE2 activator, DIZE, provides potentially a novel therapeutic approach to the treatment/prevention of atherosclerosis and fatty liver diseases by influencing macrophages polarization and taurine biosynthesis. However, the exact understanding of mechanisms of the advantageous actions of DIZE require further studies.

Nevertheless, our study has several limitations. DIZE is mainly recognized as an antitrypanosomal drug and ACE2 activator, but it also elicits other pharmacological properties. It can inhibit acid-sensitive ion channels (ASIC_1a_, ASIC_1b_, ASIC_2a_, and ASIC_3_), which play a role in the perception of pH changes during extracellular tissue acidosis [44]. Additionally, some studies showed contradictory results regarding the ability of DIZE to activate ACE2 [45,46]. However, apart from the activation of ACE2, DIZE might also increase the mRNA/protein expression of ACE2, which we also observed in our setting [20]. Further studies should be done to evaluate whether DIZE-induced elevated ACE2 activity occurs directly or indirectly as a result of increased mRNA expression.

## 4. Materials and Methods

### 4.1. Animal Studies

Twenty-two female apolipoprotein E-knockout mice on the C57BL/6J background were obtained from Taconic (Ejby, Denmark). The animals were maintained on 12 h dark/12 h light cycles at room temperature (22.5 ± 0.5 °C) and at 45–55% humidity with access to water ad libitum and diet. At the age of 8 weeks, the mice were fed with a high-fat diet (HFD) (containing 15.2% fat and 0.25% cholesterol) for 16 weeks. The diet was prepared by Morawski (Kcynia, Poland). The animals were divided into two groups: female apoE^−/−^ mice on high-fat diet (control) (*n* = 11) and female apoE^−/−^ mice on an HFD treated with diminazene aceturate (DIZE) (*n* = 11). DIZE was mixed without heating with the HFD and administered to the mice at a dose of 30 mg per kg of body weight per day. The dose of DIZE was chosen based on the previous results from mice studies [25,47]. At the age of 6 months, the mice were euthanized 5 min after injection of Fraxiparine (Nadroparin) i.p. (1000 UI; Sanofi-Synthelabo, Paris, France) in chamber filled with carbon dioxide at a rate of 20–30% CO_2_ chamber volume per minute, in accordance with AVMA Panel 2007 recommendations and institutional IACUC guidelines. The selected tissues (aortas, hearts, livers, and intestine together with mesenteric arteries) were dissected and the blood was collected. All animal procedures were conformed with the guidelines from Directive 2010/63/EU of the European Parliament on the protection of animals used for scientific purposes and were approved by the Jagiellonian University Ethical Committee on Animal Experiments (no. 67/2014).

### 4.2. Atherosclerotic Lesion Assessment

The development of atherosclerotic lesions in apoE^−/−^ mice was evaluated using cross-section method, as described before [48]. The aortic sections (10-μm thickness) were stained with Oil Red-O (Sigma-Aldrich, St. Louis, MO, USA) to measure the area of atherosclerotic plaques. The necrotic core of atherosclerotic lesions in apoE^−/−^ mice was assessed by the hematoxylin–eosin (HE) staining. Aortic images were captured using Olympus BX50 (Olympus, Tokyo, Japan) microscope, and the data were analyzed by the LSM Image Browser software (Zeiss, Jena, Germany).

### 4.3. Immunohistochemical Staining of Aortic Roots

Sections of ascending aorta were fixed in acetone and used for immunohistochemistry, as described previously [48]. To detect the content of macrophages and smooth muscles cells in atherosclerotic plaques, the sections were stained with primary antibodies against CD68 (Serotec, Kidlington, UK) (dilution 1:800) and smooth muscle α-actin (SMA) (Sigma-Aldrich, St. Louis, MO, USA) (dilution 1:800), respectively. Macrophage polarization was assessed as described before [49]. Antibodies were used against F4/80 (Abcam, Cambridge, UK) (dilution 1:100), nitric oxide synthase 2 (iNOS) (Abcam, Cambridge, UK) (dilution 1:200), arginase 1 (Abcam, Cambridge, UK) (dilution 1:100), and 4’6-diamidino-2-phenylindole (DAPI), for total macrophages, M1-like, M2-like, and cell nuclei, respectively. The images were recorded using the Camedia DP71 digital camera (Olympus, Tokyo, Japan). The analysis of all sections was performed using the LSM Image Browser software (Zeiss, Jena, Germany).

### 4.4. Histology of the Liver

The samples of the liver tissues were fixed using formalin and embedded in paraffin. The paraffin sections (2 µm thickness) were stained with hematoxylin-eosin (HE) method, as previously described [50].

### 4.5. Biochemical Measurement

The blood was centrifuged at 1000× *g* at 4 °C for 10 min, and then the plasma was collected and stored at −80 °C. The levels of total cholesterol, TG, LDL, and HDL in the plasma were measured using commercially available kits (Roche Molecular Biochemical, Pleasanton, CA, USA). In addition, levels of aspartate aminotransferase (AST) and alanine aminotransferase (ALT) were measured by the Reflovet Plus equipment (Roche, Basel, Switzerland) using commercial kits: Reflotron GOT, Reflotron GPT (Roche, Basel, Switzerland). Moreover, the Taurine Assay Kit (Cell Biolabs, San Diego, CA, USA) was used to determine the concentration of taurine in the liver. The content of TG in the liver was assayed using the Triglyceride Colorimetric Assay Kit (Cayman Chemical, Ann Arbor, MI, USA), according to the manufacturer’s guidelines.

### 4.6. Western Blot Analysis

Immunoblotting analysis used to determine the expression of CSAD was conducted as previously described [50]. Briefly, the samples were separated on SDS-polyacrylamide gels (7.5–15%) (Mini Protean II, Bio-Rad, Hercules, CA, USA) using the Laemmli buffer system and semidry transferred to nitrocellulose membranes (GE Healthcare, Chicago, IL, USA). The membranes were blocked overnight at 4 °C with 5% (*w/v*) non-fat dried milk in TTBS and incubated for 3 h at room temperature with specific primary antibodies followed by 1 h incubation with HRP-conjugated secondary antibodies (GE Healthcare, Chicago, IL, USA). Bands were developed with the use of ECL-system reagents (GE Healthcare, Chicago, IL, USA). GAPDH was used as a control of equal protein content. The following specific primary antibodies were applied: ANTI-CSAD (MyBioSource, San Diego, CA, USA) and ANTI-GAPDH (MyBioSource, San Diego, CA, USA). The images were visualized using the ImageQuant Las 500 (GE Healthcare, Chicago, IL, USA) and analyzed by Image Lite Studio software (LI-COR, Lincoln, NE, USA).

### 4.7. Real-time PCR

Real-time PCR technique was used to determine the expression levels of ACE, ACE2, and NEP genes in the aorta and the liver of apoE^−/−^ mice, and IL-1β, TNF-α, MRC1, and FCER2 genes in THP-1 macrophages according to protocol, as described previously [50]. Briefly, RNA was isolated using the RNeasy Fibrous Tissue Mini Kit (Qiagen, Hilden, Germany) and transcribed to cDNA with the High-Capacity cDNA Reverse Transcription Kit (Thermo Scientific, Waltham, MA, USA). Commercially available primers from Bio-Rad (Hercules, CA, USA) (IL-1β, TNF-α, MRC1, FCER2, and GAPDH) and Qiagen (Hilden, Germany) (ACE, ACE2, NEP) and GoTaq^®^ qPCR Master Mix (Promega, Madison, WI, USA) were used to carry out the real-time PCR reaction. Analysis of relative gene expression with GAPDH as an internal reference gene was performed by the 7900HT fast real-rime PCR System (Applied Biosystems, Foster City, CA, USA), and the data were analyzed using the 2^−∆∆C*t*^ method by Data Assist v3.01 software (Applied Biosystems, Foster City, CA, USA).

### 4.8. Mesenteric Arteries Preparation

Segment of intestine together with mesenteric arteries was quickly excised from mice and placed in cold saline solution. The segment of first- or second-order branch of the superior mesenteric artery was cleared from surrounding adipose tissue and cannulated in the pressure myograph (JP Trading, Aarhus, Denmark). The chamber of the pressure myograph as well interior of vessel was filled with modified Krebs-Henseleit solution having following composition in mM: NaCl 119, KCl 4.7, KH_2_PO_4_ 1.18, MgSO_4_ 1.17, CaCl_2_ 2.5, NaHCO_3_ 25, glucose 5.5, pyruvate 2, and EDTA 0.5. The buffer in the chamber was bubbled with gas mixture of 21% oxygen and 5% carbon dioxide with nitrogen and temperature was set at 37 °C. The outer diameter of the vessels was continuously monitored by a video camera attached to an inverted microscope. After 30 min of stabilization at 10 mm Hg, pressure was raised to 60 mm Hg and stabilized for another 15 min. All drugs were applied extraluminally to the myograph chamber. The experiment protocol was as follows: after stabilization, concentration–response curve for phenylephrine (Phe) (in the range of 10^−7^ to 10^−5^ M) was obtained. After washing with Krebs-Henseleit buffer, vessel was submaximally preconstricted with Phe (usually 10^−6^ M), and increasing concentrations of acetylcholine (Ach) (also in the range of 10−^7^ M to 10^−5^ M) were applied. Next, similar concentration–response curve for DEA-NO was obtained. Then, after washing, but without preconstriction with Phe, increasing doses (in the range of 10^−9^ to 10^−6^ M) of angiotensin II were applied. Last substance tested was KCl in the concentration range of 30–90 mM. Finally, passive diameter was measured after incubating vessel in calcium-free Krebs-Henseleit buffer. The relaxation response was expressed as a percentage of the pre-contraction induced by phenylephrine, and the EC50 values for individual vessels were calculated.

### 4.9. Proteomics Studies in the Liver

Liver samples from apoE^−/−^ mice and apoE^−/−^ mice treated with DIZE (*n* = 4 per group) were homogenized using a Tissue Lyser LT (Qiagen, Germany) and lysed in a buffer containing 0.1 M Tris-HCl, pH 8.0, 2% sodium dodecyl sulfate, and 50 mM dithiothreitol (Sigma Aldrich, Saint Louis, MI, USA) at 96 °C for 10 min. Protein concentration was measured by Pierce 660 nm Protein Assay Kit (Thermo Scientific, USA). Seventy micrograms of protein content were digested using the multiple enzyme digestion filter aided by a sample preparation method (MED FASP) [51,52] with two enzymes: endoproteinase LysC and trypsin. Next, samples were purified with C18 MacroSpin Columns (Harvard Apparatus, Cambridge, MA, USA) and prepared as recommended by the iTRAQ protocol (AB Sciex, Framingham, MA, USA). Four samples from each group were labeled with iTRAQ reagents as follows: control—113, 115, 117, 119 and DIZE—114, 116, 118, 121. Then, the labeled samples were combined, dried in a vacuum concentrator (Eppendorf, Hamburg, Germany), and dissolved in 0.1% trifluoroacetic acid in order to purify it with C18 MacroSpin columns (Harvard Apparatus, Cambridge, MA, USA). Eluates were reconstituted in 0.2 ammonium formate, pH 10.0, and subject to fractionation under high pH conditions (Harvard Apparatus, Cambridge, MA, USA). Peptides were eluted in 10 consecutive salt steps (15%, 17.5%, 20%, 22.5%, 25%, 27.5%, 30%, 32.5%, 35%, and 50% acetonitrile in 0.05 M ammonium formate) and dried in a vacuum concentrator. The samples were dissolved in 5% acetonitrile with 0.1% formic acid and concentrated on a trap column (Acclaim PepMap100 RP C18 75 µm i.d. × 2 cm column, Thermo Scientific Dionex, Sunnyvale, CA, USA) and then injected on-line onto a PepMap100 RP C18 75 µm i.d. × 15 cm column (Thermo Scientific Dionex, Sunnyvale, CA, USA). Peptides were separated over a 90 min 7–55% B phase linear gradient (A phase: 2% acetonitrile and 0.1% formic acid; B phase: 80% acetonitrile and 0.1% formic acid) with a flow rate of 300 nL/min by UltiMate 3000 HPLC system (Thermo Scientific Dionex, Sunnyvale, CA, USA) and applied on-line to a Velos Pro (Thermo Scientific, Waltham, MA, USA) dual-pressure ion-trap mass spectrometer. The nano-electrospray ion source (Nanospray Flex, Thermo Scientific, Waltham, MA, USA) parameters consisted of ion spray voltage of 1.7 kV and capillary temperature of 250 °C. The spectra were collected over a full scan mode (400–1500 Da) followed by one higher energy collisional dissociation (HCD) of the five most intense ions from the preceding survey’s full scan under dynamic exclusion criteria. These spectra were analyzed by the X!Tandem (The Global Proteome Machine Organization) and Comet search algorithms and then validated with Peptide Prophet and iProphet under Trans-Proteomic Pipeline software (Institute for Systems Biology, Seattle, WA, USA). Search parameters consisted of several aspects: (1) taxonomy: rat (UniProtKB/Swiss-Prot); (2) enzyme: trypsin; (3) missed cleavage sites allowed: 2; (4) fixed modification: Methylthio(C); (5) variable modifications: methionine oxidation(M); (6) iTRAQ 8-plex (K), iTRAQ 8-plex (N-term), and iTRAQ 8-plex (Y); (7) parent mass error: 1.5 to + 3.0 Da; and (8) peptide fragment mass tolerance: 0.7 Da. Quantitative information was extracted with Libra software under Trans-Proteomic Pipeline. The peptide false discovery rate was estimated by Mayu (Trans-Proteomic Pipeline), and peptide identifications with false discovery rates < 1% were considered as correct matches. DanteR software was used for statistical analysis of iTRAQ-labeled peptides [53]. Briefly, the data were log2 transformed and normalized using quantile regression. Analysis of variance (ANOVA) was performed at a peptide level and the Benjamini and Hochberg false discovery rate (FDR) correction was used to adjust *p*-values. The mass spectrometry proteomic data were deposited to the ProteomeXchange Consortium via the PRIDE partner repository with the dataset identifier PXD022829.

### 4.10. THP-1 Cell Culture

Human THP-1 monocytic cell line (ATCC, Manassas, VA, USA) was grown in a humidified incubator containing 5% CO_2_ and 95% air at 37 °C in RPMI 1640 medium (Gibco, MA, USA) supplemented with 10% fetal bovine serum (FBS, Gibco, MA, USA) and streptomycin (100 μg/mL)/penicillin (100 U/mL). In order to differentiate THP-1 monocytes to macrophages, the cells were placed in 6-well plates (1.5 × 10^6^ cells per well, passage 1–3) in 3 mL of culture medium and treated with 10 nM phorbol 12-myristate 13-acetate (PMA; Sigma Aldrich, St. Louis, MO, USA) for 72 h. After 3 days of resting, THP-1 macrophages were polarized for 24 h with 100 ng/mL LPS (*Salmonella Minnesota*; InvivoGen, San Diego, CA, USA) or 33 ng/mL IL-4 (R&D Systems, Minneapolis, MN, USA) to M1 and M2 macrophages, respectively. DIZE (10 μM, based on our previous results) was added 1 h before stimulation with LPS and IL-4. The expression of M1 and M2 markers was assessed using real-time PCR.

### 4.11. Statistical Analysis

The data were expressed as a mean + SEM. The equality of variance (F-test) and the normality of the data (Shapiro–Wilk test) were checked and based on the outcome. The statistical analysis was performed using nonparametric Mann–Whitney U test or *t*-test/one-way ANOVA with multiple comparisons and Benjamini and Hochberg false discovery rate (FDR) correction (GraphPad Prism 8, San Diego, CA, USA). The values of *p* < 0.05 were considered statistically significant.

## 5. Conclusions

We have shown that ACE2 activator, DIZE, given orally for 16 weeks, was able to stabilize atherosclerotic lesions and attenuate hepatic steatosis in apoE^−/−^ mice fed an HFD. Such effects were associated with decreased total macrophages content but increased anti-inflammatory M2 macrophages and α-smooth muscle actin levels in atherosclerotic plaques. Interestingly, the anti-steatotic action of DIZE in the liver was related to the decreased levels of triglycerides in liver, elevated levels of HDL in the plasma, and increased biosynthesis and concentration of taurine. Yet, the exact molecular mechanisms of both the anti-atherosclerotic and anti-steatotic actions of DIZE require further clarifications.

## Figures and Tables

**Table 1:** Differentially expressed proteins in the liver of apoE^−/−^ mice treated with DIZE as compared to the control group (*p* < 0.05, *n* = 4 per group).

UniProtKB ID.	Gene Name	Protein Name	Fold Change
Q91Z98	Chil4/Ym2	Chitinase-like protein 4	3.36
Q9DBE0	Csad	Cysteine sulfinic acid decarboxylase	1.45
P20152	Vim	Vimentin	1.37
P07724	Alb	Serum albumin	1.35
P19157	Gstp1	Glutathione S-transferase P 1	1.26
Q80W21	Gstm7	Glutathione S-transferase Mu 7	1.26
P02088	Hbb-b1	Hemoglobin subunit beta-1	1.25
P19639	Gstm3	Glutathione S-transferase Mu 3	1.25
O35728	Cyp4a14	Cytochrome P450 4A14	1.25
P07356	Anxa2	Annexin A2	1.22
P10649	Gstm1	Glutathione S-transferase Mu 1	1.22
P97371	Psme1	Proteasome activator complex subunit 1	1.20
Q9D379	Ephx1	Epoxide hydrolase 1	1.15
P80315	Cct4	T-complex protein 1 subunit delta	1.15
Q6P3A8	Bckdhb	2-oxoisovalerate dehydrogenase subunit beta, mitochondrial	1.15
P48036	Anxa5	Annexin A5	1.13
Q921I1	Tf	Serotransferrin	1.13
Q923D2	Blvrb	Flavin reductase (NADPH)	1.12
P24456	Cyp2d10	Cytochrome P450 2D10	1.12
Q9EQK5	Mvp	Major vault protein	1.12
P62806	Hist1h4a	Histone H4	1.12
P62737	Acta2	Actin, aortic smooth muscle	1.10
P15105	Glul	Glutamine synthetase	1.09
P54869	Hmgcs2	Hydroxymethylglutaryl-CoA synthase, mitochondrial	1.09
P27773	Pdia3	Protein disulfide-isomerase A3	1.07
P26443	Glud1	Glutamate dehydrogenase 1, mitochondrial	1.07
P11725	Otc	Ornithine carbamoyltransferase, mitochondrial	−1.04
Q9R0H0	Acox1	Peroxisomal acyl-coenzyme A oxidase 1	−1.06
P05784	Krt18	Keratin, type I cytoskeletal 18	−1.06
Q8C196	Cps1	Carbamoyl-phosphate synthase [ammonia], mitochondrial	−1.06
P24270	Cat	Catalase	−1.06
Q9WVL0	Gstz1	Maleylacetoacetate isomerase	−1.06
Q61176	Arg1	Arginase-1	−1.07
P50247	Ahcy	Adenosylhomocysteinase	−1.08
P30115	Gsta3	Glutathione S-transferase A3	−1.09
Q8BVI4	Qdpr	Dihydropteridine reductase	−1.09
P61922	Abat	4-aminobutyrate aminotransferase, mitochondrial	−1.10
P35492	Hal	Histidine ammonia-lyase	−1.10
Q9QXF8	Gnmt	Glycine N-methyltransferase	−1.10
P52840	Sult1a1	Sulfotransferase 1A1	−1.11
Q8R0Y6	Aldh1l1	Cytosolic 10-formyltetrahydrofolate dehydrogenase	−1.12
P62631	Eef1a2	Elongation factor 1-alpha 2	−1.14
O35490	Bhmt	Betaine-homocysteine S-methyltransferase 1	−1.14
P49429	Hpd	4-hydroxyphenylpyruvate dioxygenase	−1.15
O88428	Papss2	Bifunctional 3′-phosphoadenosine 5′-phosphosulfate synthase 2	−1.16
Q91X83	Mat1a	S-adenosylmethionine synthase isoform type-1	−1.17
P05201	Got1	Aspartate aminotransferase, cytoplasmic	−1.20
P16460	Ass1	Argininosuccinate synthase	−1.22
P29758	Oat	Ornithine aminotransferase, mitochondrial	−1.25

## Data Availability

The mass spectrometry proteomic data were deposited to the ProteomeXchange Consortium via the PRIDE partner repository with the dataset identifier PXD022829.

## Abbreviations

ACE	angiotensin-converting enzyme
ACE2	angiotensin-converting enzyme 2
ALT	alanine aminotransferase
AST	aspartate aminotransferase
CSAD	cysteine sulfinic acid decarboxylase
DIZE	diminazene aceturate
HDL	High-density lipoprotein
HE	hematoxylin/eosin
HFD	High-fat diet
LDL	low-density lipoprotein
NAFLD	nonalcoholic fatty liver disease
NEP	neprilysin
RAS	renin-angiotensin system
TG	triglyceride
